# Social connectedness and loneliness in school for autistic and allistic children

**DOI:** 10.1177/13623613241259932

**Published:** 2024-06-18

**Authors:** Yung-Ting Tsou, Maedeh Nasri, Boya Li, Els M A Blijd-Hoogewys, Mitra Baratchi, Alexander Koutamanis, Carolien Rieffe

**Affiliations:** 1 Leiden University, The Netherlands; 2 Vrije Universiteit Amsterdam, The Netherlands; 3 University of Groningen, The Netherlands; 4 INTER-PSY, The Netherlands; 5 Delft University of Technology, The Netherlands; 6 University of Twente, The Netherlands; 7 University College London, UK

**Keywords:** autism, individual differences, loneliness, school climate, social connectedness

## Abstract

Autistic children are often reported less socially connected, while recent studies show autistic children experiencing more loneliness in school than allistic (i.e. non-autistic) children, contradicting the traditional view that autistic children lack social motivation. This study aimed to understand individual differences in how social connectedness is construed, between and within groups of autistic and allistic pupils, using a multimethod approach. Forty-seven autistic and 52 neurodiverse-allistic classmates from two special primary schools participated (8–13 years). Proximity sensors worn by pupils on playgrounds during recess measured (1) total time in face-to-face contacts, (2) number of contact partners, and (3) centrality in playground networks. Peer reports measured (4) reciprocal friendships and (5) centrality in classmate networks. To evaluate their *feelings* of connectedness, pupils rated the level of loneliness in school. Compared with allistic pupils, autistic pupils had fewer reciprocal friendships, but similar total time in social contacts, number of partners, classmate/playground centrality, and levels of loneliness. Lower levels of loneliness related to higher classmate centrality in autistic children, but longer time in social contacts in allistic children. For these autistic children, being *liked* as part of a peer group seems essential. Understanding relevant differences in children’s needs could lead to a more welcoming school climate.

**Lay abstract:**

Many previous studies reported that autistic children have fewer social connections. Yet, recent studies also show that autistic children more often feel lonely in school than allistic (i.e. non-autistic) children. This outcome seems to go against the traditional view that autistic children do not desire to have social connections. Therefore, this study aimed to find out how autistic and allistic children feel about their social connections. We included 47 autistic and 52 neurodiverse-allistic children from two special education primary schools (aged 8–13 years). We tested their social connections and loneliness in school, through a new approach. This new approach includes questionnaires, and sensors for tracking social contacts on playgrounds during school breaks. We found that allistic children felt more loneliness when they spent little time in social contacts during school breaks. Yet, autistic children felt more loneliness when their peers did not like to play with them. For these autistic children, feelings of loneliness may go beyond face-to-face contacts. Being liked as part of a peer group was key. Understanding differences in children’s needs can lead to a more effective design for a welcoming school climate.

School is the main setting where most children experience social interactions outside the family circle. For a child to learn and practice social skills, these social interactions are crucial. But the nature of these interactions also relates to how socially connected a child feels at school (Allen et al., 2018; Craggs & Kelly, 2018). When there is a mismatch between the desired and actual amount of social connectedness—that is, when a child is cognitively aware of the unmet desire in the quality and quantity of their social connections—feelings of loneliness arise (Asher & Paquette, 2003). Feelings of loneliness in school can be stressful and painful, and they can contribute to mental health problems, for example, symptoms of depression and low self-esteem (Cacioppo et al., 2006; Santini et al., 2021). Yet, what is less well-known is the extent to which individual children may differ in how they construe social connectedness, especially among autistic children. For example, an extensive literature has suggested that autistic pupils lack the desire to build social connections (Chevallier et al., 2012; Dawson, 2008; Klin et al., 2003). Yet, other studies have found elevated levels of loneliness in school among autistic adolescents, compared with allistic (i.e. non-autistic) peers, thereby challenging that view (Bauminger & Kasari, 2000; Deckers et al., 2017; also see Bottini, 2018; Jaswal & Akhtar, 2019 for arguments against the “social motivation” view). These discrepancies reflect a gap in the literature regarding individual differences in how autistic and allistic pupils construe their own social connectedness.

In this study, we focused on groups of primary-school autistic and allistic pupils, and distinguished two types of social connectedness: (1) physical connectedness, that is, pupils’ physical proximity with their peers in school at recess, and (2) emotional connectedness, that is, the peer connections with which pupils identify. Furthermore, we examined how pupils *felt* about their social connectedness, by measuring their subjective feelings of loneliness. To capture the social dynamics in the school environments, we uniquely employed a multimethod approach that combined self-report, peer nomination, and wearable sensor technology.

## Social connectedness of autistic pupils

Pupils may build social connections simply by being in proximity, such as playing next to a peer on the playground. Such opportunities for physical contact alone would suffice to promote peer interaction (Rademaker et al., 2020) and foster mutual understanding (see “contact theory”; Allport, 1954). This type of *physical connectedness* in school could be particularly relevant for autistic pupils, as it may allow them to remain part of the group and learn social skills without becoming overwhelmed by the social demands required for building more intimate relationships. Despite this, current empirical evidence from observations shows that autistic pupils experience fewer physical contacts in primary school compared with allistic peers. They more often spend time alone during recess, engaging in unoccupied or solitary activities, and initiate or respond to social interactions less frequently (Anderson et al., 2004; Chang et al., 2019; Falkmer et al., 2015; Locke et al., 2016).

When a social connection becomes meaningful to pupils and is acknowledged (Bowles & Scull, 2019; Phares, 1993), a psychological bond and preferences may form among them, leading to a sense of *emotional connectedness*. Past research has shown that when asked to identify their social connections, autistic pupils tend to receive fewer nominations from peers as social group members, often occupying more peripheral positions in a peer network, and engage in fewer reciprocal friendships, compared with their allistic peers (e.g. Bottema-Beutel et al., 2019; Chamberlain et al., 2007; Kasari et al., 2011; Locke et al., 2013, 2016). In addition, they are less often perceived as “someone to hang out with” by allistic peers (Chamberlain et al., 2007), and are more often considered as not preferred across their primary school years (Locke et al., 2013). This pattern seems to primarily apply to autistic boys, who are more frequently rejected, while autistic girls are more often overlooked (i.e. not mentioned in any types of nominations; Dean et al., 2014). These findings touch upon the idea that autistic pupils may feel less emotionally connected to their peers in school.

However, different results also surfaced when the perspectives of autistic pupils themselves were involved. Reports show that autistic pupils in primary school perceive themselves as socially involved because they nominate more friends and “buddies” than their allistic peers, although these nominations are more often unreciprocated (Chamberlain et al., 2007). At the same time, qualitative evidence indicated that many autistic pupils reported having one friend and being satisfied with the friendship (Calder et al., 2012). For these autistic pupils, qualities such as shared interests, trust, and companionship seemed more important in their peer relationships, compared with the other qualities like reciprocity and closeness that were often valued by allistic pupils (Calder et al., 2012; O’Hagan & Hebron, 2016; Sedgewick et al., 2016; Vine Foggo & Webster, 2017; also see Cresswell et al., 2019 for a review). Learning from autistic pupils’ varying experiences in school is thus crucial.

## Loneliness in autistic pupils

While it appears that primary-school autistic children are lower in their *physical* and *emotional* connectedness in school than are allistic peers, the question is, “Are these children alone but satisfied with their level of social connection, or are they experiencing an unmet need and feeling lonely?” To the best of our knowledge, only three studies directly compared levels of loneliness between autistic and allistic pupils in primary schools, via standardized self-report questionnaires, and no group differences were reported (aged 7–11 years; Bottema-Beutel et al., 2019; Chamberlain et al., 2007; Deckers et al., 2017). This is likely because autistic pupils at this age do see themselves as socially involved, as discussed above (Chamberlain et al., 2007). Moreover, feelings of loneliness were found to be unrelated to children’s overall friendship quality, or to how prominent they were in a peer group, both in autistic and in allistic children (Bottema-Beutel et al., 2019; Chamberlain et al., 2007).

Nevertheless, in studies that also included adolescents, self-reported levels of loneliness in autistic participants were consistently higher than in their allistic peers (Bauminger et al., 2003; Bauminger & Kasari, 2000; Chang et al., 2019; Deckers et al., 2017; Locke et al., 2010; Whitehouse et al., 2009). In adolescence, most pupils experience a transition in their social environment, where peers become the primary partners in daily social interaction (Berndt, 1982; Von Salisch, 2001). This transitional period can be particularly challenging for autistic adolescents given the heightened expectations from the social environment.

Notably, different factors seem to be relevant to these adolescents’ reported loneliness. For allistic adolescents, higher levels of loneliness were related to fewer intimate and prosocial interactions with peers, while these relations were not observed in autistic adolescents (Bauminger et al., 2003; Bauminger & Kasari, 2000; Chang et al., 2019). Rather, autistic adolescents felt lonelier when their social networks did not provide a sense of togetherness and safety, that is, when they experienced lower levels of trust and companionship in their friendships, and more limited school participation (Bauminger & Kasari, 2000; Chang et al., 2019). Thus, when we want to gain a better understanding of the factors affecting loneliness in autistic youth, we need to consider possible individual differences, and an approach that can capture dynamic features for social connectedness.

## Present study

Peer interaction is essential in most children’s school life. But how do they feel about the connections that result from this interaction, and do differences such as being autistic lead to different ways of viewing these connections? Such questions are important to answer, as they extend our knowledge on how children’s social well-being in school may be enhanced.

In this study, we aimed to assess differences in how social connectedness was construed, both between and within groups of primary-school autistic and allistic pupils, respectively. We distinguished between *physical* and *emotional* social connectedness, and examined their relationships with pupils’ feelings of loneliness, to understand the potential sources of unmet social connectedness needs. To this end, we recruited two special education schools, a setting where autistic children were with other neurodivergent peers in their class and where their needs are better addressed, compared with most mainstream schools investigated in many prior studies (e.g. Anderson et al., 2004; Bottema-Beutel et al., 2019; Chamberlain et al., 2007; Falkmer et al., 2015; Kasari et al., 2011; Locke et al., 2013). Moreover, we adopted a multimethod approach that included self-report, peer report, and wearable sensor technology (Radio Frequency Identification Devices, or RFID). RFID has been shown to reveal social dynamics among children during school recess in an objective and unobtrusive manner (Cattuto et al., 2010; Nasri et al., 2022; Veiga et al., 2017).

**Figure 1.**
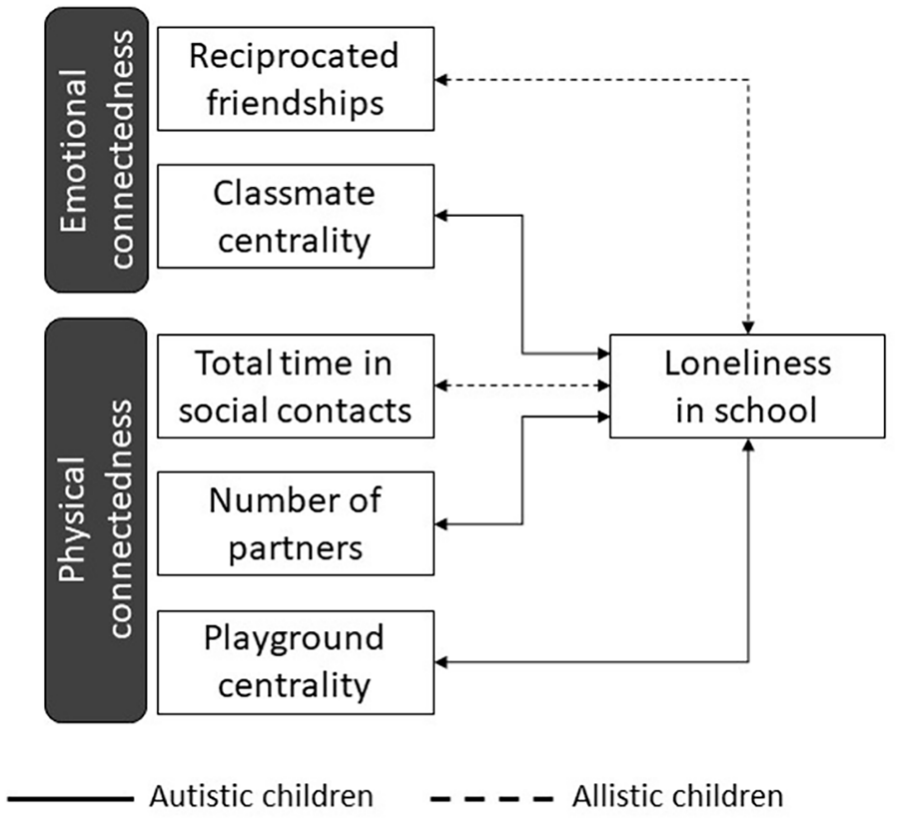
Overview of the study variables and hypotheses. Solid lines represent the hypotheses for the autistic group; dotted lines represent the hypotheses for the allistic group. The double-headed arrows denote the negative correlations expected between physical/emotional and felt social connectedness.

With this set-up, first, we aimed to determine the levels of *physical* and *emotional* connectedness (Figure 1). RFID data revealed children’s *physical* connectedness on the playground during school recess. From the RFID data, we computed (1) amount of time spent in face-to-face social contacts, (2) each child’s number of contact partners, and (3) their level of connectedness to the entire playground social network (i.e. the degree of “centrality” that reflects how physically close each child was to all the other peers in the playground social network). For *emotional* connectedness, peer nominations were used to measure reciprocity in friendships, and each child’s relative level of connectedness to the class social network (i.e. the degree of centrality that reflects how all the other classmates in the class network were emotionally available to each child). By including the centrality measure, we took into account pupils’ connectedness to the larger peer network. Given that autistic pupils may value their social connections differently from their allistic peers (e.g. Bauminger & Kasari, 2000; Chang et al., 2019), we considered both the stronger and weaker social connections. We expected autistic pupils to be less connected than allistic pupils, as measured by peer nomination and by objective data collected in RFID sensors at recess, although this prediction was based on prior studies including autistic children in mainstream school settings. We also expected to find significant variance among individual scores, on all measures.

Second, we aimed to understand *how children felt* about their social connectedness at school. Therefore, we examined feelings of loneliness in school to understand the extent to which children were unsatisfied with their current level of social connectedness; and investigated the extent to which children’s loneliness was related to their *physical* and *emotional* connectedness with peers, according to the above measures. We expected no difference in the levels of loneliness between autistic and allistic pupils, as the pupils in this study were still in primary-school years (Bottema-Beutel et al., 2019; Chamberlain et al., 2007; Deckers et al., 2017). Furthermore, previous studies with relevant age groups showed that more loneliness was associated with less intimate and less positive peer relationships among allistic pupils, whereas for autistic children, more loneliness was associated with a lack of opportunities to safely be part of the school activities (Bauminger et al., 2003; Bauminger & Kasari, 2000; Chang et al., 2019; Whitehouse et al., 2009). Therefore, we expected that allistic pupils would feel less lonely when they have more reciprocated friendships, and/or when they spent more time in face-to-face contacts; and autistic pupils would feel less lonely when they were included in school social activities more often, for example, when they had contact with more peers during recess and/or occupied a more central position within their peer groups. However, this part of the hypothesis was exploratory in nature.

## Methods

### Participants

All pupils in this study attended two primary schools for special education in the Netherlands (School A and School B). Autistic pupils (*n* = 47) and their allistic classmates (*n* = 52), aged 8 to 13 years, were recruited to participate (*M*_age_ = 10.84 years, *SD* = 1.21; 34 girls and 65 boys). The participation rate was 68% for School A and 73% for School B among all the pupils in the age range in the schools. In School A the majority of pupils were allistic, that is, without an autism diagnosis, while in School B the majority had an autism diagnosis (see Table 1). Nineteen (40%) of the autistic pupils had additional diagnoses related to psychiatric/behavioral conditions, such as attention deficit hyperactivity disorder (ADHD). Among the allistic pupils, 18 (35%) had a diagnosis of psychiatric/behavioral conditions that did not involve autism (hence, hereafter this group of pupils is referred to as “neurodiverse-allistic classmates” for clarity; see Table 1 for more details about the distribution of these diagnoses). The autistic group was younger (*t*(96) = 5.41, *p* < 0.001) and had fewer girls and more boys (χ^2^ = 22.30, *p* < 0.001) than the neurodiverse-allistic group. Note that specific data on socioeconomic status were not recorded.

**Table 1.** Background characteristics of the participants.

	Autistic	Neurodiverse-allistic
*N*	47	52
Age, years, *M* (*SD*)	10.20 (1.00)	11.38 (1.14)
Gender, *n* (%)		
Girls	5 (11)	29 (56)
Boys	42 (89)	23 (44)
School distribution, *n* (%)		
School A	6 (13)	46 (88)
School B	41 (87)	6 (12)
Playgroup allocation, *n* (%)		
Lower grades (5–6)	27 (57)	27 (52)
Higher grades (7–8)	20 (43)	26 (48)
Additional psychiatric/behavioral conditions, *n* (%)		
None or unknown	6 (13)	35 (67)
Autism only	24 (51)	–
Attention Deficit Hyperactivity Disorder (ADHD)	17 (36)	16 (31)
Developmental Language Disorder	2 (4)	1 (2)
Oppositional Defiant Disorder	0	1 (2)

In the Netherlands, special education is divided into four clusters (1: low vision; 2: serious communication difficulties, for example, hearing loss or language disorder; 3: cognitive/physical disabilities or a chronic illness; and 4: psychiatric or serious behavioral difficulties, for example, autism, ADHD, and/or oppositional defiant disorder (ODD)). In this study, both autistic and neurodiverse-allistic children were recruited from two Cluster 4 schools. Table 1 shows that 51% of the autistic children had no additional psychiatric/behavioral disorders, whereas 67% of the neurodiverse-allistic children had no diagnosis related to psychiatric/behavioral conditions. Also, both the autistic and neurodiverse-allistic groups had similar proportions of pupils with ADHD (36% and 31%, respectively). Besides, both schools accepted several pupils without a specific diagnosis who switched from mainstream schools due to difficulties in adjusting to the pace of learning, class size, and peer interactions, and their needs to receive extra care and support. The special education setting provides activities that are more structured and predictable, and gives more personal attention and specialist support to individual students. Both schools are members of the same private educational organization, using similar teaching methods, and structuring their school activities and rules similarly. During recess, children shared the playground with peers from their grade; teachers supervised the recess time but did not intervene in the activities unless necessary.

Before a child can be admitted to a Cluster 4 school, the receiving school must request a declaration of admissibility from the regional education council (i.e. the governmental organization responsible for the management of the education in the region). The council is obliged to be advised by at least two experts (from a committee of remedial educationalists, child psychologists/psychiatrists, social workers, and doctors) for verifying the condition and issuing an admissibility statement. Based on this system, in this study we asked for the diagnosis information from parents, and confirmed these with their teachers according to the school documents.

This study was part of a large-scale research project that examines different aspects of social participation and inclusion of autistic children in schoolyards (e.g. Nasri et al., 2022, 2023). Guardians of child participants signed informed consent forms prior to test procedures. The study protocol and informed consent form were approved by the Psychology Research Ethics Committee of Leiden University, the Netherlands.

### Measures and procedures

Children completed self- and peer-report questionnaires on a tablet, accompanied by either their teacher in the classroom or an experimenter in a separate room in school. Before filling out the questionnaires, they were presented an instruction video on the tablet, which described the purpose of the study, instructed how to fill out the questionnaires, and showed that they can ask questions. Teachers and experimenters were instructed to only provide support when necessary, that is, when children asked questions or when they appeared to have misunderstood the questions.

Sensor data were collected from each child on four occasions on two school days, during morning and lunch recess time on both days, each lasting 11 to 30 min (*M* = 18.97 min, *SD* = 6.62). Before recess, all pupils were given a belt they wore on their waist, on which an RFID tag was mounted, facing front. Pupils were explained that they could take off the belt when they were not comfortable with it, but only one to two children in 2% of the break sessions did that. They wore the belt throughout recess on the playground, and returned the belt when the recess ended. Teachers on the playground were also given a sensor belt, although in this study only the social contacts between pupils were considered. During recess on the playground, children were not given specific instructions regarding where or with whom to play.

#### Measures of physical connectedness via wearable proximity sensors

OpenBeacon RFID tags (OpenBeacon, n.d.) are proximity sensors by means of Bluetooth, registering face-to-face contact between pupils on the playground during recess. RFID is an unobtrusive and objective measure that allows for quantifying spatial proximity between children in their daily school settings, continuously throughout a recess. It does not intervene children’s behavior and ensures ecological validity (Veiga & Rieffe, 2018). Previous research has proven the accuracy and specificity of the RFID technology, showing that the social contacts detected by the RFID tags corresponded to video observations and self-reported amount of social contacts, in both adults and children (Elmer et al., 2019; Veiga et al., 2017). Moreover, RFID tags have been previously used in autistic children and deaf and hard-of-hearing children (Eichengreen et al., 2024).

Pupils on the playground were each given an RFID tag mounted to a belt, facing front. A signal-receiving base station captured RFID signals from an area covering 15 m^2^ four times per second (Veiga & Rieffe, 2018). It was located on the school playground at a predetermined location to ensure maximized detection range. When two children, while wearing an RFID tag, were facing each other and within a distance of 1.5 m, the tags detected Bluetooth signals, and passed on the data to the signal-receiving base station, which then registered a social contact. RFID tags could detect multiple contacts simultaneously. To compensate for unintended interruptions, contacts with interruptions shorter than 35 s were registered as one single contact (Cattuto et al., 2010; Nasri et al., 2022). During recess, some children may move to areas that were out of the detection coverage of the base station, for example, because they went inside the building or to the toilet. To ensure a fair comparison between all children, we only considered the RFID records of a participant when the participant was detected by the base station for at least 50% of the recess time. Otherwise, the data for that participant in that specific recess session were excluded from further analyses. Sensor data points from the four measurements (two breaks × two days) were averaged for further analyses.

**Figure 2.**
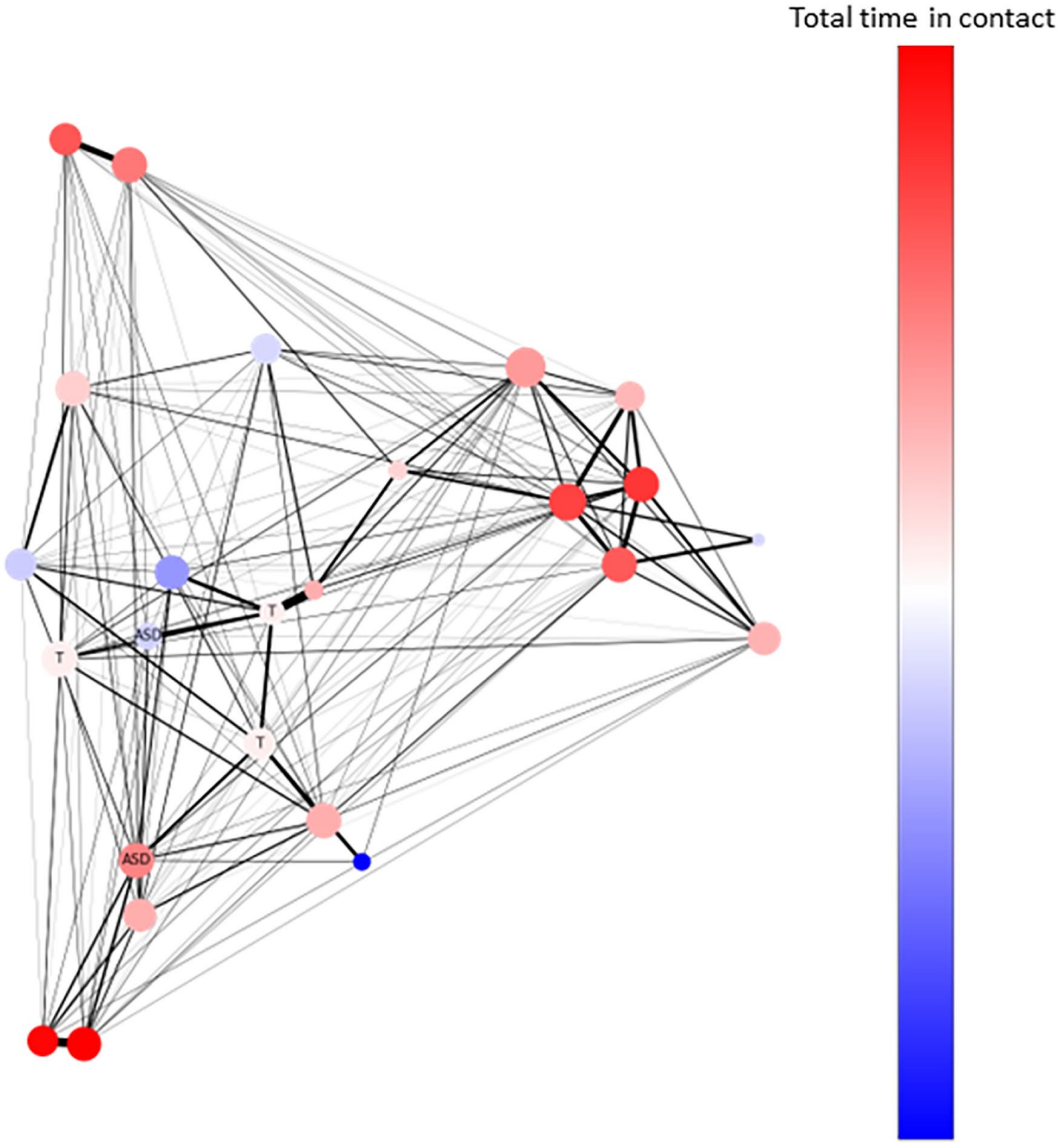
Visualization of a sample social network detected by the proximity sensors (RFID) during one school recess session. Each node represents an individual on the playground. Autistic children are labeled with “ASD”; teachers with “T.” The color of the nodes denotes total time in social contacts during this recess session; warmer colors (red vs. blue) suggest longer time. The thickness of the edges between two nodes denotes the duration of dyadic contacts; thicker edges suggest two notes/children having longer contacts with each other. The nodes are positioned in accordance with their centrality.

We included three variables derived from social networks detected by the RFID (Figure 2): *Total time in social contacts* indicated the proportion of time a participant spent in face-to-face contacts during recess. This was calculated by dividing the total duration of time spent throughout all contacts by the total duration of time that a participant was detected by the base station, in a specific recess session.

*Number of contact partners* indicated the number of different peers that a participant had social contact with during recess. This was calculated by dividing the total number of contact partners by *N* – 1, where *N* denotes the total number of pupils detected on the playground in a specific recess session.

Finally, we calculated *playground closeness centrality* from the RFID data to examine each pupil’s connectivity to all the other peers on the playground. The closeness centrality was computed according to the formula C(u)=n−1∑v−1n−1d(v,u) (Freeman, 1979), where *d*(*v*, *u*) denotes the shortest-path distance between Child *v* and Child *u*, and *n* denotes the total number of participating children on the playground. Here, we further weighed this score by dyadic contact time measured by the RFID, to reflect how closely a participant was connected to the network, both in terms of the shortest-path distance to reach all the other peers in the network, and the time spent with them. Dyadic contact time refers to the duration of contact between two children, normalized by the total duration when both children were detected by the receiving station, in a specific recess session. The inverse of this value was used as the weight; thus, longer dyadic contact time led to a smaller weight (i.e. a shorter path), which led to a higher playground closeness centrality. Therefore, this playground closeness centrality measure combined both the time and the number of partners in each social contact, reflecting individual children’s relative position in their playground social network (Freeman, 1979; Zhang & Luo, 2017): A higher playground closeness centrality in this study thus indicated that the participant was more physically close to all the other peers on the playground than those with a lower closeness centrality.

#### Measures of emotional connectedness via peer nomination

To examine *reciprocated friendship*, peer nominations were obtained by asking each child to write down the names of their best friends in school. They could provide a maximum of five names. This limitation of maximally five names ensured that we obtained stronger social connections that can be recalled by the children, following Pijl et al. (2008). From these nominations, we derived the number of nominations given by each participant (i.e. *outdegree* in social network analysis) and the number of nominations that were reciprocated (i.e. *bi-degree*; the participant nominated a peer as a friend, and the peer also nominated back). We computed the degree of reciprocated friendships by dividing *bi-degree* by *outdegree*.

To examine each pupil’s connectedness to their larger peer network in the class, each participant was presented with a list of classmates, and they answered the extent to which they liked to play with each classmate (i.e. “yes,” “sometimes,” “no,” or “I don’t know”). Based on peers’ ratings, we calculated a *classmate closeness centrality* score for each participant. A higher classmate closeness centrality score indicated that the participant could approach all the other peers in the class more easily—more likely to be “liked”—than those with a lower centrality (Zhang & Luo, 2017). When Child v receives a “yes” or “sometimes” rating directly from Child u, the two children are seen as having a connection with an one-unit distance. Based on all the classmate ratings in each class, their centrality scores were computed using the same formula described above. Here, we treated both “sometimes” and “yes” answers as indicating being part of the social network, but “sometimes” answers were weighted with a distance of 1, while “yes” answers were weighted with a distance of 0.5 (i.e. a shorter path). Only the ratings given by peers were considered.

#### Loneliness in school via self-reports

Children’s Loneliness Scale (CLS) was used to assess levels of loneliness in school in terms of children’s dissatisfaction with social connections (Asher & Wheeler, 1985; validated in Dutch-speaking children: Maes et al. (2017)). This is a self-report consisting of 24 items, rated on a 5-point scale (1 = *not at all*, 5 = *always*). Six items that were positively formulated were reverse scored, thus higher scores indicating higher levels of loneliness. Eight items were control items about children’s hobbies and preferred activities, and were excluded from further analyses. Internal consistency was good (Cronbach’s α = 0.87 for all children; 0.87/0.88 for autistic/neurodiverse-allistic pupils).

### Statistical analyses

Closeness centrality from peer reports was computed using *igraph* within R (Csardi & Nepusz, 2006; R Core Team, 2023). Variables derived from the RFID data were preprocessed and computed using Python 3.9 (Van Rossum & Drake, 1995). The *NetworkX* 2.6.3 Python package was used for visualization. Statistical analyses were performed using SPSS version 27.0 (SPSS Inc., Chicago, IL, USA).

First, to examine the extent to which autistic and neurodiverse-allistic children differed in their connectedness in social networks (*physical* and *emotional* connectedness) and loneliness, a series of Mann–Whitney *U* tests were conducted. Rank-based nonparametric tests were used due to the presence of outliers in the variables of loneliness, playground closeness centrality, and classmate closeness centrality. To assess whether the variance among individual scores was equivalent between the two groups, Levene’s tests of homogeneity of variances, based on the deviation from the median values, were used. Next, to examine the extent to which feelings of loneliness were related to connectedness in social networks, partial Spearman’s correlation tests were administered, controlling for age. Fisher’s r-to-z transformation was used to examine the moderating role of autism, comparing the strength of correlation between autistic and neurodiverse-allistic children. To correct for multiple testing, the Bonferroni procedure was applied, and the significance level of the main analyses was adjusted to *p* < α/5 = 0.01.

Little’s MCAR test showed that data were missing completely at random (χ^2^ = 177.82, *p* = 0.111). Thus, we handled missing data using the multiple imputation (MI) technique (Azur et al., 2011; Schafer & Graham, 2002; Van Ginkel et al., 2020). Ten imputations were performed.

Given the age and gender differences between autistic and neurodiverse-allistic pupils, and to reduce the effect of the school of origin, inverse probability of treatment weighting (IPTW) procedure was applied (Austin & Stuart, 2015; Chesnaye et al., 2022; Ruth et al., 2015). IPTW is a widely used weighting method for adjusting confounding variables. Following the procedures proposed by Chesnaye et al. (2022), first the probability of a participant being in the autistic versus neurodiverse-allistic group was computed into a propensity score, taking into account individuals’ characteristics (i.e. age, gender, and school). Next, a weight was assigned to each participant, computed as the inverse of the propensity score, through which potential confounds were balanced across groups. To avoid extreme weights that may bias the outcomes, the weights were stabilized by accounting for the proportion of autistic versus neurodiverse-allistic children, and extreme values beyond the first and 99th percentiles were truncated.

Pooled and weighted results were reported. Correlations between all study variables are presented in Supplemental Appendix A. Results based on raw data are reported in Supplemental Appendix B. To understand the effect of participant heterogeneity, we also ran the analyses while excluding the autistic children with comorbidity and neurodiverse-allistic children with a diagnosis (Supplemental Appendix C), and compared social connectedness between autistic children with and without comorbidity (Supplemental Appendix D).

### Community involvement

The overall objectives of the larger project were formulated in meetings with autistic self-advocates and researchers, as well as practitioners working with autistic individuals. Also, the new methodology involving sensing technologies was discussed with associations for promoting the interests of autistic people, school organizations, and governmental organizations, although they were not directly engaged in formulating the research questions addressed in this study.

## Results

### Levels of physical and emotional connectedness and loneliness

Table 2 shows the mean levels and standard deviations for the study variables. Regarding the observed levels of social connectedness as measured by peer reports and RFID, autistic children had fewer reciprocated friendships (*U* = 1528.0, *p* = 0.002) than neurodiverse-allistic children. There were no group differences in total time in social contacts, in the number of contact partners, or in classmate/playground closeness centrality (*U*s ⩾ 1878.0, *p*s ⩾ 0.145). Regarding the levels of loneliness, no group differences were noted (*U* = 1904.50, *p* = 0.181).

**Table 2.** Mean levels and standard deviations of social connectedness and the Spearman’s correlations with loneliness (controlling for age).

	Range	Mean (*SD*)			Correlation with loneliness		
		Autistic	Neurodiverse-allistic	*U*	All	Autistic	Neurodiverse-allistic
Loneliness (total score^a^)	16-68	33.38 (8.03)	37.03 (11.11)	1904.5	–	–	–
Physical connectedness							
Total time in social contact^b^	0.03–1	0.62 (0.22)	0.63 (0.20)	1878.0	−0.06	0.30	−0.39***
Number of contact partners^c^	0.09–0.95	0.56 (0.15)	0.56 (0.15)	2002.5	−0.08	–	–
Playground closeness centrality	0.02–0.11	0.08 (0.02)	0.08 (0.02)	1892.0	0.12	–	–
Emotional connectedness							
Reciprocated friendships^d^	0–1	0.39 (0.25)	0.48 (0.24)	1528.0**	0.04	–	–
Classmate closeness centrality	0.47–2	1.05 (0.34)	1.12 (0.34)	2091.5	−0.10	−0.36**	0.08

*Note*. Correlation coefficients for separate groups are reported only when Fisher’s r-to-z transformation showed a significant difference in the strength of correlations between the group; otherwise, the correlation coefficients for the entire sample are reported.

a Highest possible total score is 80.

b Corrected by the total time when the child was detected.

c Corrected by *n* – 1, where *n* is the total number of children on the playground.

d Calculated as a degree by dividing the number of reciprocated nominations by the number of outgoing nominations.

** *p* ⩽ 0.01. ***p ⩽ 0.001.

Tests of homogeneity of variances showed that variances for the loneliness scores were not equal between the two groups (*SD*_autistic_ = 8.03 < *SD*_allistic_ = 11.11, *F*(1, 93) = 5.04, *p* = 0.027). For the other variables, the variances were equivalent across the groups, *F*s < 1.60, *p*s > 0.208.

### Relations between social connectedness and loneliness

Higher classmate closeness centrality was related to lower loneliness only in autistic children (ρ = –0.36, *p* = 0.004), not in neurodiverse-allistic children. More time spent in social contacts was related to lower loneliness only in neurodiverse-allistic children (ρ = –0.39, *p* < 0.001), not in autistic children. No other significant correlations or group differences in correlational strength were noted (Table 2).

## Discussion

The present study aimed to examine how autistic children construed their social connectedness in school, compared with their neurodiverse-allistic classmates. In line with our expectations, autistic pupils in this study had fewer reciprocated friendships than neurodiverse-allistic pupils. Unexpectedly, autistic and neurodiverse-allistic children were similarly connected to their peers, in terms of time spent in social contacts and number of interaction partners during recess, and their centrality in classmate and playground networks. The levels of loneliness experienced by the two groups at school did not differ. However, the factors related to their loneliness did differ: While neurodiverse-allistic children felt lonelier when they spent less time in physical social contact during recess, autistic children reported higher loneliness when they were less central, that is, less liked as a classmate to play with, in the classmate network. No other relations were noted.

Unlike many previous studies that reported autistic children were less connected to their peers than allistic children (e.g. Anderson et al., 2004; Chamberlain et al., 2007; Falkmer et al., 2015; Kasari et al., 2011; Locke et al., 2013, 2016), in this study we found comparable outcomes in most aspects of physical and emotional connectedness, including total time in social contacts, number of contact partners during recess, and their centrality in peer networks. That is, we observed that autistic children were in social contacts at the group level to the same extent as their neurodiverse-allistic peers.

It could be argued that this positive picture was likely a result of the special education setting where we collected data. That is, compared with settings in mainstream education, autistic pupils in special education are usually not the only ones with a diagnosis; class sizes in special education are smaller; school activities including recess are more structured; and teachers are better equipped with skills to identify problems and support and facilitate positive social dynamics among children (Blatchford et al., 2011; De Swart et al., 2021; Lane et al., 2005; Sokal & Sharma, 2013). In fact, in our sample, autistic children were the majority in several of the classes. In this context, autistic children have more opportunities to meet their autistic peers and other peers with a special need. When it is recognized that all pupils have their unique needs, being “different” with a diagnosis and social difficulties may be less of an issue for joining peer activities (Florian, 2014). Moreover, it gives autistic children an environment to connect with other autistic and neurodivergent peers who may understand them better, share similar interests, and experience similar challenges in certain social situations. They may also feel more relaxed, not having to live up to allistic social norms and to mask themselves constantly (Heasman & Gillespie, 2018). Our study provided a unique opportunity to further our understanding of autistic-to-autistic/neurodivergent interactions, and showed that such a context can be highly positive for autistic children’s connectedness to peers, which they can hardly achieve in mainstream schools unless in a self-contained setting. A more structured recess in special education settings may also allow more face-to-face contacts to be facilitated and thus be detected.

Although beyond the scope of the current study, our findings suggest that the school climate and how the school environment is organized may play an important role in children’s social participation, beyond individual children’s diagnosis and social skills. Several prior studies have also shown that autistic children become more socially engaged when the school playground was adapted to provide more equitable opportunities that also address autistic children’s needs and capacities, for example, by reducing noises and improving acoustic to lower overstimulation commonly encountered by autistic pupils in school settings, by offering more structure (e.g. by making different compartments with different functions), and by addressing different sensory needs (e.g. to set up different sensory zones and transition between the zones; see Harris et al. (2024), Mostafa (2014), Rieffe and Koutamanis (2023), and Yuill et al. (2007)).

Our use of sensor technology may also have contributed to such findings. The present study showed the first attempt to capture social dynamics between autistic children and their peers throughout recess, in a naturalistic setting, objectively and unobtrusively (see Nasri et al. (2022) and Veiga and Rieffe (2018) for more information about this methodological approach). This method returns objective and richer information regarding children’s group dynamics, complementing methods in previous studies (e.g. systematic observation), which can be constrained by observation timeframe, observer bias, and the coding scheme.

However, it seems that many autistic children were not considered a friend by peer group members, and had fewer reciprocated friendships than neurodiverse-allistic peers. The peer-report measure for reciprocated friendships was the only social connectedness indicator in this study that required active responses from peers, and it denoted the reciprocity perceived by peers, toward a specific child. To receive more reciprocated friendship nominations in a free-recall task, a child has to be prominent enough in a network for the other peers to select them as a friend. In such a scenario, it appears that autistic children were more often overlooked by their social group members.

The question then is, “How did the autistic children feel, in light of their lack of connectedness?” Our results showed that the degree of reciprocated friendships was unrelated to feelings of loneliness, that is, there was no mismatch between the reciprocated friendships that autistic children desired and perceived. Possible explanations for this include that (1) autistic children were not particularly aware of reciprocity in their social connections; (2) they did not care about or want many connections, or acted like they did not care for self-protection; or (3) they did belong to a social group, despite not having many apparent connections (Chamberlain et al., 2007). Adding to the study by Chamberlain and colleagues (2007), our results seem to substantiate the third explanation. In this study, the large majority of autistic children did have at least one peer who liked to play with them (*n* = 44; 94%), and they also experienced that they had at least one friend (*n* = 38; 81%), which might meet their needs to know that they are not alone. Moreover, in autistic children, more loneliness was related to being less liked in a classmate network (i.e. lower classmate closeness centrality, an aspect of *emotional* connectedness), whereas in neurodiverse-allistic children more loneliness was related to spending less time in social contacts during recess (an aspect of *physical* connectedness). Apparently, for these autistic children, being liked as part of a social group, experiencing group-level emotional connectedness, is closely related to their feelings of loneliness. It is thus likely that in autistic children, social connectedness is not evaluated based on dyadic contacts, but beyond that, based on the extent that one feels accepted by a group. When they do feel they belonged to a social group, the risk of them feeling lonely might decrease.

These results highlight the importance of looking into individual differences and widening the possible definition of social relationships. Different individuals could find different features of social connection to be valuable. While spending time together may be seen as a relationship goal by many people, it may not always be the case for others. Actually, in autistic children, total time in social contacts during recess had a positive, rather than negative, correlation with loneliness (ρ = 0.30; although not reaching the significance level after the Bonferroni correction). Possibly, having to stay in face-to-face interaction with others could cause stress, anxiety, and exhaustion in autistic children, as they must constantly attend to social cues that they may not fully understand, and/or some may still feel the need to camouflage or hide their social difficulties so as to “fit in” (Black et al., 2024; Corbett et al., 2014; Dean et al., 2017). These challenges can be further aggravated in adolescence due to the even less structured environment—moving from one classroom to another, going to school cafeteria (i.e. the place for the lunch break in school), and no supervision during recess—which may underlie autistic adolescents’ elevated levels of loneliness in school as reported by previous studies (e.g. Bauminger & Kasari, 2000; Deckers et al., 2017; Whitehouse et al., 2009). Nevertheless, despite the possible differences in how social features were viewed, the fact that autistic children experienced loneliness in school at varying levels, to an extent that was comparable with that of allistic children, shows that autistic children do value social connection and are aware of unfulfilled desires for social relationships, like their allistic peers.

### Limitations and future research

This study was among the first to examine *physical* and *emotional* connectedness in autistic children and their relations with loneliness in school, using a multimethod approach that accounted for social dynamics over an entire recess session. Yet, some limitations should be taken into account, and some caution is due when interpreting the results.

First, as mentioned earlier, all participants were from special education schools and most allistic pupils also had a diagnosis, although not autism. We were thus able to investigate what the social situation is like when autistic pupils are substantially represented in a class network. This may explain why the measured levels of social connectedness were largely similar across the groups, while previous studies in mainstream schools (where usually only one or two autistic pupils were present) showed lower levels in all aspects (e.g. Anderson et al., 2004; Chamberlain et al., 2007; Kasari et al., 2011; Locke et al., 2013, 2016). Future studies could explore the extent to which these outcomes are generalizable to mainstream settings, and how the findings from special education settings may inform inclusive practices in other settings. Future research is also required to understand how the school climate may vary with different school policies for inclusion and diversity, and how an improved school climate may further enhance children’s social well-being. Focus group interviews could be organized with children belonging to different groups, to take a variety of views into account.

Here, our finding regarding the importance of group-level emotional connectedness—a sense of belonging to a group—among autistic pupils requires special attention. This may become increasingly difficult to achieve as their social challenges intensify during adolescence. This may (partially) explain the higher levels of loneliness found in autistic adolescents when compared with their allistic peers (Bauminger & Kasari, 2000; Bauminger et al., 2003; Chang et al., 2019; Deckers et al., 2017; Locke et al., 2010; Whitehouse et al., 2009). Moreover, this may imply that school policies and practices could be especially influential for the school life of these pupils.

Second, half of the autistic children in this study had comorbidity (besides their diagnosis of autism; see Mannion and Leader (2013) for a review on comorbidity in autism), and 35% of the neurodiverse-allistic children had at least one diagnosis. Given the Cluster 4 special education setting, the majority of the participants were not neurotypical. These sample characteristics should be taken into account, as they influenced the social dynamics. Future studies are encouraged to confirm whether the patterns we found for the neurodiverse-allistic group is specific to this sample that included many pupils with ADHD and several pupils who transitioned from mainstream education. However, we included all children regardless of their diagnosis in the study, because our main goal was to examine the effect of autism on social connectedness. Our findings highlighted how autistic and neurodiverse-allistic pupils may have different social needs, which should be considered when providing support.

Notably, in our follow-up analyses, where we excluded autistic children with comorbidity and neurodiverse-allistic children with a diagnosis, we confirmed the same differential patterns (Supplemental Appendix C). However, after those children with comorbidities were removed, autistic and neurodiverse-allistic children no longer differed in the number of reciprocated friendships. Further inspection added that autistic pupils with comorbidities had fewer reciprocal friends, while contacting more partners—hence spending shorter time with each partner—during recess, compared with autistic pupils without any comorbidities (Supplemental Appendix D). While these outcomes might be affected by the smaller sample size, it is likely that comorbidity could put autistic children in a more vulnerable position, regarding the formation of close relationships. Future studies are needed to further understand the needs and wishes for social connectedness of children with comorbidities or other diagnoses, for creating a school environment where all children are respected.

Third, the two groups differed in several aspects, besides their diagnoses. The autistic group was older and featured fewer girls than the neurodiverse-allistic group, and most of the autistic children were from one school in the study, while most of the neurodiverse-allistic children were from the other school. While the IPTW method can balance the characteristics in the samples with reliable outcomes, effectively reducing the impact of confounding effects, there could still be potential biases in the results that we overlooked. Moreover, it should also be noted that due to the distribution of autistic versus neurodiverse-allistic pupils in the two schools, autistic children were more likely to choose an autistic peer as friends and had contact with them compared with neurodiverse-allistic children. Although the aim of this study was not to examine autistic children’s intergroup interaction, but to understand their social connectedness in a setting where they were not the only ones “different” with a diagnosis, our findings should be interpreted with caution. Despite the fact that the two schools we selected were managed by the same educational organization and organized school activities in a largely similar way, there might be factors in the two schools that influenced our results. To further assist interpretation of our findings, results based on raw data were also presented (Supplemental Appendix B). Whether weighted or not, our results consistently revealed group differences in reciprocated friendships, and in the relation between social contact time and loneliness in allistic children, but not in autistic children.

Fourth, some limitations in our data collection should be noted. In this study, the Children’s Loneliness Scale (Asher & Wheeler, 1985) was used to examine levels of loneliness in terms of children’s unmet needs of social connections. Yet, it should be noted that this measure has not been formally validated among autistic children. Prior studies have shown that autistic children may define loneliness differently from their allistic peers: They tended to focus on the dissatisfaction of social connections, whereas allistic children more often also mentioned the associated negative affect (Bauminger & Kasari, 2000). This possible discrepancy should thus be considered in future studies, and we also call for further research to validate existing loneliness measures in autistic youth separately. Also, peer nominations were limited to the school setting, yet it is likely that children also have connections outside of school, for example, in the neighborhood. Furthermore, the proximity sensors, that is, the RFID badges, captured only face-to-face contacts within 1.5 m. Social contacts are not always on a face-to-face basis, or within such a close distance. Children in a playground may play together side-by-side (which is often observed in autistic children; Gunn et al., 2014; Holmes & Willoughby, 2005) or talk to each other from a distance, but those interactions could be largely missed with the current configuration. Moreover, these detected social contacts may not necessarily reflect social engagement, as pupils may be in close proximity but not involved in a joint activity. The governmental measures such as social distancing in response to the COVID-19 pandemic might also affect the social dynamics between children to some extent, although at the time of our data collection, no constraints were imposed.

## Conclusions

Most children go to school on a daily basis. Understanding how they feel and how to promote their well-being in school is of utmost importance. Such knowledge, however, is limited in the literature, especially regarding children with special needs, who compose at least 10% of the student population (United Nations Children’s Fund, 2021). Our findings provide evidence that loneliness in school may be construed differently by autistic and neurodiverse-allistic children, although the levels of loneliness were comparable in the two groups. For these autistic children, feelings of loneliness may go beyond face-to-face interactions. Rather, being liked as part of a peer group was key.

Our findings call for further investigations that examine individual differences in social connectedness, and for school-based interventions that move the focus from individual children’s social skills, for the purpose of “fitting into” peer activities, to adapting the school environment so it promotes inclusion. Understanding relevant differences in children’s needs could well lead to more effective design for a welcoming school climate. This in turn could increase the social well-being of not only autistic children, but all children at school.

## Supplemental Material

sj-docx-1-aut-10.1177_13623613241259932 – Supplemental material for Social connectedness and loneliness in school for autistic and allistic children Supplemental material, sj-docx-1-aut-10.1177_13623613241259932 for Social connectedness and loneliness in school for autistic and allistic children by Yung-Ting Tsou, Maedeh Nasri, Boya Li, Els M A Blijd-Hoogewys, Mitra Baratchi, Alexander Koutamanis and Carolien Rieffe in Autism
