# Supplementary material for: Social connectedness and loneliness in school for autistic and allistic children
Source: Autism. 2024 Jun 18;29(1):87–101. doi: 10.1177/13623613241259932 (PMC11656637; doi:10.1177/13623613241259932)
Supplement: sj-docx-1-aut-10.1177_13623613241259932 – Supplemental material for Social connectedness and loneliness in school for autistic and allistic children [file sj-docx-1-aut-10.1177_13623613241259932.docx]

**Appendix A.** Correlations between the study variables (pooled and weighted results).

|  | Spearman’s rho in all children (in autistic / neurodiverse-allistic children) | | | | | |
| --- | --- | --- | --- | --- | --- | --- |
|  | 2. | 3. | 4. | 5. | 6. | 7. |
| 1. Age | **-.275**  (-.052**/-.423**) | **.211**  (.220/.129) | **-.369**  (-.177/-**.445**) | **.248**  (.282/.211) | **-.239**  (-**.298**/-.210) | .088  (.015/.113) |
| 2. Loneliness |  | -.019  (.101/-.193) | .009  (-**.344**/.256) | -.128  (.268/-**.437**) | -.008  (-.035/.079) | .093  (.204/.020) |
| 3. Reciprocated friendships |  |  | .079  (.152/.092) | **.367**  (**.349**/**.346**) | .072  (.154/.105) | .194  (.273/.133) |
| 4. Classmate centrality |  |  |  | **-.311**  **(-.367**/-.236) | **.276**  (.159/**.356**) | -.133  (-.285/.015) |
| 5. Time in social contact |  |  |  |  | **.231**  (.282/.206) | **.495**  (**.692**/**.317**) |
| 6. N contact partners |  |  |  |  |  | **.727**  (**.742**/**.735**) |
| 7. Playground centrality |  |  |  |  |  |  |

*Note*. Significant correlations (p ≤ .01) are bolded.

**Appendix B.** Results based on raw data (unweighted and before multiple imputation).

|  |  | Mean (SD) | | | Correlation with loneliness | | |
| --- | --- | --- | --- | --- | --- | --- | --- |
|  | Range | Autistic | Neurodiverse-allistic | *U* | All | Autistic | Neurodiverse-allistic |
| Loneliness (total score^a^; *n* = 85) | 16-68 | 32.63 (10.02) | 33.30 (11.92) | 898.5 | - | - | - |
|  |  |  |  |  |  |  |  |
| Physical connectedness |  |  |  |  |  |  |  |
| Total time in social contact^b^ (*n* = 83) | 0.03-1 | .54 (.23) | .70 (.23) | 522.0** | -.14 | .11 | -.41 |
| Number of contact partners^c^ (*n* = 83) | 0.09-0.95 | .60 (.17) | .53 (.17) | 666.0 | -.12 | - | - |
| Playground closeness centrality (*n* = 83) | 0.02-0.11 | .07 (.02) | .08 (.02) | 741.0 | -.02 | - | - |
|  |  |  |  |  |  |  |  |
| Emotional connectedness |  |  |  |  |  |  |  |
| Reciprocated friendships^d^ (*n* = 76) | 0-1 | .34 (.29) | .52 (.30) | 459.5** | -.03 | - | - |
| Classmate closeness centrality (*n* = 75) | 0.47-2 | 1.20 (.36) | .94 (.35) | 433.5** | -.11 | - | - |

*Note*. Correlation coefficients for separate groups are reported only when Fisher’s r-to-z transformation showed a significant difference in the strength of correlations between the group; otherwise, the correlation coefficients for the entire sample are reported.

^a^ Highest possible total score is 80.

^b^ Corrected by the total time when the child was detected.

^c^ Corrected by *n* – 1, where *n* is the total number of children on the playground.

^d^ Calculated as a degree by dividing the number of reciprocated nominations by the number of outgoing nominations.

** *p* ≤ .01. *** *p* ≤ .001.

**Appendix C.** Results including only autistic children without comorbidity (*n* = 24) and neurodiverse-allistic children without a diagnosis (*n* = 34) (weighted and pooled results).

|  |  | Mean (SD) | | | Correlation with loneliness | | |
| --- | --- | --- | --- | --- | --- | --- | --- |
|  | Range | Autistic | Neurodiverse-allistic | *U* | All | Autistic | Neurodiverse-allistic |
| Loneliness (total score^a^) | 16-68 | 33.32 (6.96) | 35.99 (14.42) | 687.50 | - | - | - |
|  |  |  |  |  |  |  |  |
| Physical connectedness |  |  |  |  |  |  |  |
| Total time in social contact^b^ | 0.03-0.99 | .65 (.23) | .65 (.24) | 600.00 | -.07 | .35 | -.44** |
| Number of contact partners^c^ | 0.11-0.95 | .53 (.14) | .54 (.15) | 693.50 | -.05 | - | - |
| Playground closeness centrality | 0.02-0.11 | .08 (.02) | .08 (.02) | 546.00 | .15 | - | - |
|  |  |  |  |  |  |  |  |
| Emotional connectedness |  |  |  |  |  |  |  |
| Reciprocated friendships^d^ | 0-1 | .46 (.26) | .49 (.28) | 602.00 | -.10 | .28 | -.36 |
| Classmate closeness centrality | 0.49-2 | 1.02 (.38) | .91 (.23) | 533.50 | -.20 | -.43** | .23 |

*Note*. Correlation coefficients for separate groups are reported only when Fisher’s r-to-z transformation showed a significant difference in the strength of correlations between the group; otherwise, the correlation coefficients for the entire sample are reported.

^a^ Highest possible total score is 80.

^b^ Corrected by the total time when the child was detected.

^c^ Corrected by *n* – 1, where *n* is the total number of children on the playground.

^d^ Calculated as a degree by dividing the number of reciprocated nominations by the number of outgoing nominations.

** *p* ≤ .01. *** *p* ≤ .001.

**Appendix D.** Mean and standard deviation of the study variables in autistic children with comorbidities (*n* = 23) and autistic children without comorbidities (*n* = 24) (weighted and pooled results).

|  | Autistic children  with comorbidities | | Autistic children without comorbidities | | *U* | *p* |
| --- | --- | --- | --- | --- | --- | --- |
|  | Mean | SD | Mean | SD |  |  |
| Loneliness | 33.51 | 10.25 | 33.32 | 6.96 | 450.00 | .932 |
| Reciprocated friendships | .24 | .18 | .46 | .26 | 238.50 | **.002** |
| Classmate closeness centrality | 1.12 | .24 | 1.02 | .38 | 415.00 | .554 |
| Total time in social contact | .56 | .20 | .65 | .23 | 407.00 | .478 |
| Number of contact partners | .62 | .15 | .53 | .14 | 295.50 | .020 |
| Playground closeness centrality | .08 | .02 | .08 | .02 | 416.00 | .564 |
